# Relationship between a novel non–insulin-based metabolic score for insulin resistance (METS‐IR) and coronary artery calcification

**DOI:** 10.1186/s12902-022-01180-7

**Published:** 2022-11-10

**Authors:** Zhenwei Wang, Xiaofang Hui, Xu Huang, Jingjie Li, Naifeng Liu

**Affiliations:** 1 Department of Cardiology, Zhongda Hospital, School of Medicine, Southeast University, Nanjing, China; 2 Department of Emergency Medicine, Zhongda Hospital, School of Medicine, Southeast University, Nanjing, China; 3 Department of Hematology and Oncology, Affiliated Xuchang People’s Hospital of Xinxiang Medical College, Xuchang, China

**Keywords:** Insulin resistance, Metabolic score for insulin resistance, Triglyceride glucose index, Coronary artery calcification

## Abstract

**Background and aims:**

A novel non–insulin-based metabolic score for insulin resistance (METS‐IR) index has been proposed as a simple and reliable alternative insulin resistance (IR) marker, but its the predictive value in asymptomatic adults with coronary artery calcification (CAC) remains unclear.

**Methods and results:**

We enrolled 1576 participants without cardiovascular disease (CVD), who underwent multidetector computed tomography. Logistic regression, restricted cubic spline models and receiver operating characteristic (ROC) curves were used to examine the association between METS-IR, the ratio of triglyceride to high-density lipoprotein cholesterol (TG/HDL-C) and triglyceride glucose index (TyG index) and CAC. In multivariate logistic regression analysis, the increase in METS-IR was independently associated with a higher prevalence of CAC (all P < 0.05 in Models 1–3). Furthermore, restricted cubic splines indicated that the significance of METS-IR in predicting CAC was higher than that of other IR indexes. In ROC curve analysis, without considering the P value, the area under the curve of CAC predicted by METS-IR was higher than that of other IR indexes (METS-IR, 0.607; TyG index, 0.603; TG/HDL-C, 0.577).

**Conclusion:**

Compared with other IR indexes, METS-IR may have better discrimination ability in predicting the incidence of CAC in asymptomatic adults without CVD.

## Introduction

At present, cardiovascular disease (CVD) has been established as the main cause of morbidity and mortality all over the world, which has greatly increased the physical and economic burden on people [1]. Over the years, although secondary prevention strategies for CVD including drug therapy, revascularization and rehabilitation have been broadly developed and applied, the risk of recurrence and mortality of cardiovascular complications is still comparatively high in patients with CVD, especially those with high risk factors [2]. Therefore, the primary prevention of CVD is particularly important, such as identifying and correcting risk factors in high-risk groups of CVD. Coronary atherosclerosis has been recognized as the leading cause of CVD, so timely detection and prevention of coronary atherosclerosis has become the main strategy for the primary prevention of CVD. Coronary artery calcification (CAC) is a highly reliable and robust biomarker for coronary atherosclerosis and is closely related to the traditional risk factors for CVD, which plays an important role in the primary prevention of CVD [3–5]. Several studies have unanimously indicated that CAC assessed by multidetector computed tomography (CT), an imaging technique used to noninvasively quantify coronary calcium, can be used as a reliable and repeatable predictor and prognostic factor for CVD, independent of other traditional risk factors [4, 6, 7]. Current guidelines also show that screening for CAC is very important to improve cardiovascular risk assessment and guide the use of preventive treatment in the middle-and low-risk populations without CVD and asymptomatic individuals [8, 9]. Therefore, it is very important to identify CAC and its risk factors if we want to develop new therapeutic targets and customize risk reduction strategies that match individual risk levels.

Insulin resistance (IR), a component of metabolic syndrome, has been shown to be associated with a high risk of a variety of diseases, including diabetes, hypertension, obesity and coronary artery disease (CAD) [10]. Pathophysiological studies have shown that IR promotes hyperglycemia, dyslipidemia and a proinflammatory state, which may be the main reason for the progression of CAC [11]. In addition, IR is not only the cause of up to 40% of myocardial infarction, but also the most important single risk factor for CAD in young adults [12]. At present, there are a variety of methods to assess IR. First, euglycaemic-hyperinsulinaemic clamp (EHC) as the gold standard for the evaluation of IR was first proposed in the 1970s, and then it was widely used, but it was limited in large-scale clinical studies and epidemiological investigations because of its laborious, expensive, complex and time-consuming characteristics [13]. Subsequently, the homeostasis model assessment for IR (HOMA-IR) derived from fasting insulin and glucose levels was first reported in the 1980s and has been shown to have a strong correlation with IR assessed by EHC [14]. HOMA-IR is the most widely used method to assess IR when fasting insulin data are available. However, because fasting insulin is not an indicator of routine measurement, its application in some studies is also limited. In view of this, some researchers have developed several alternative indicators of noninsulin–based IR, such as the ratio of triglycerides to high-density lipoprotein cholesterol (TG/HDL-C) and the triglyceride glucose index (TyG index), which are easier to obtain and cheaper in clinical practice [15, 16]. Since the TyG index was developed to evaluate IR, a large number of studies have shown that TyG, a surrogate marker closely related to HOMA-IR, could independently predict the occurrence and prognosis of hypertension, diabetes, stroke and other CVD, even better than HOMA-IR [17–19]. However, IR is related not only to glucose and lipid metabolism, but also to nutritional status and body fat distribution. Therefore, when evaluating metabolic related diseases, these two markers of IR, which do not include nutritional components and body fat distribution, may also have certain defects.

Therefore, Bello-Chavolla OY et al. developed a new non–insulin-based metabolic score for IR (METS‐IR) in 2018, and showed that METS-IR was a very valuable score for assessing cardiac metabolic risk in healthy and high-risk subjects, as well as a promising screening tool for IR [20]. In addition, they also indicated that METS-IR was better than the TyG index and TG/HDL-C in the diagnosis of type 2 diabetes in Mexican participants, which might be because the formula of METS-IR included not only glucose and lipid metabolism indicators, but also nutritional indicators, which also increased the degree of fit between METS-IR and EHC [20]. Since the development of METS-IR, some studies have shown that METS-IR is associated with adipokine disorder, inflammatory activity, arterial stiffness, hypertension, diabetes and ischemic heart disease [20–26]. IR is very important in the occurrence and development of CVD, but there are a variety of assessment tools for IR, and invasive or expensive assessment tools in clinical settings are limited, so it is very meaningful to find a noninvasive, low-cost and effective assessment tool for IR in clinical settings. However, the relationship between METS-IR and CAC has not yet been reported. Based on this, to fill this knowledge gap and identify more effective IR assessment methods in clinical settings, the present study was designed with the aims of: (1) identifying the potential association between METS-IR and CAC; and (2) determining whether METS-IR has better discrimination ability in predicting the incidence of CAC in asymptomatic adults without CVD, compared with other non–insulin‐based IR indexes.

## Subjects, materials and methods

### Study population

**Fig. 1:**
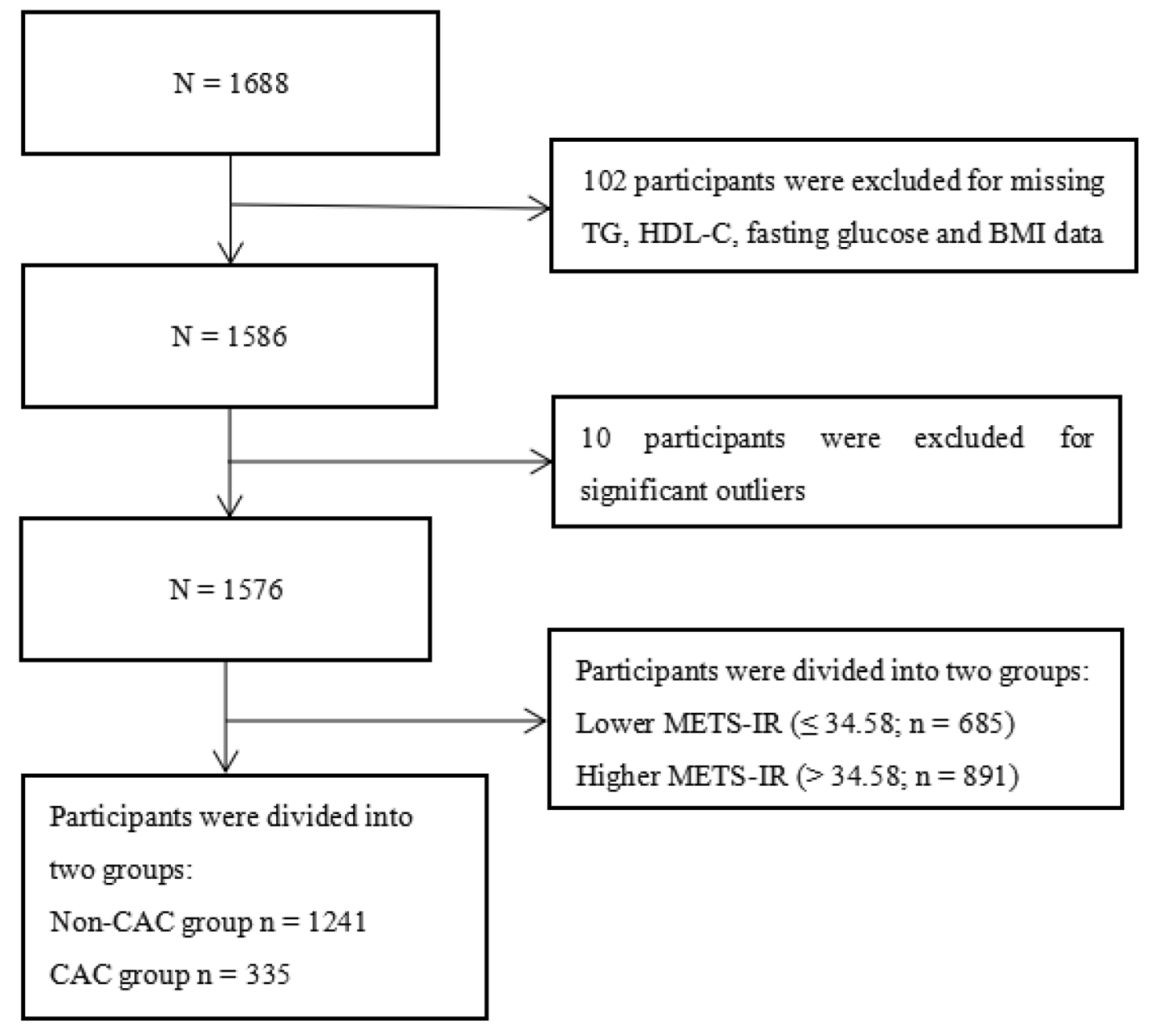
Flow chart of the study participants. *TG* triglyceride, *HDL-C* high-density lipoprotein cholesterol, *BMI* Body mass index, *METS-IR* metabolic score for insulin resistance, *CAC* coronary artery calcification

The present study was a secondary analysis based on a cross-sectional study performed by Choi SY et al. at the Seoul National University Hospital Healthcare System Gangnam Center from January 2014 to March 2016, they aimed to use a genome-wide association study (GWAS) among 1688 participants to explore single nucleotide polymorphisms (SNPs) that may be associated with severe CAC in an asymptomatic Korean population, more details can be found elsewhere [27]. Participants with a history of CVD and symptoms of CAD and participants with missing TG, HDL-C, fasting glucose, body mass index (BMI) and CAC measurements were excluded. Ultimately, 1576 participants were finally enrolled in the current study (Fig. 1). The original study protocol was approved by the ethics committee of the Seoul National University Hospital. Informed consent was waived owing to the retrospective nature of the study, and the study was carried out in accordance with the principles of the Declaration of Helsinki as revised in 2008.

### Data collection and definitions

Data from the present study were obtained from a free public database (https://figshare.com), which allowed researchers to download and use original data. After the authors of the original research shared the data, the raw data were protected by the data sharing policy, so we were free to use the data for secondary analysis without harming the rights and interests of the authors. However, when using these data, we needed to cite data sources [28]. In the original data file, the data variables needed for the present study were as follows: age, sex, smoking, diabetes, hypertension, BMI, systolic blood pressure (SBP), diastolic blood pressure (DBP), fasting glucose, total cholesterol (TC), TG, low-density lipoprotein cholesterol (LDL-C), HDL-C, high sensitivity C-reactive protein (Hs-CRP), creatinine, hemoglobin Alc (HbA1c) and CAC score (CACS). The details of the collection and measurement of the above data have been described elsewhere [27].

In this study, smoking status was divided into two groups: none or past and current. BMI was calculated as weight (kg)/height (m)^2^. Diabetes was defined as fasting blood glucose ≥ 126 mg/dl, HbA1c ≥ 6.5%, or taking hypoglycemic agents, or history of diabetes diagnosis. Hypertension was defined as SBP/DBP ≥ 140/90 mmHg or taking antihypertensive agents or history of hypertension diagnosis. The noninsulin-based markers of IR were calculated based on previously reported formulas, as follows: the TyG index was calculated as ln(fasting glucose [mg/dL] × fasting TG [mg/dL]/2) [15], and the METS-IR was determined as ln(2 × fasting glucose [mg/dL] + fasting TG [mg/dL]) × BMI [kg/m^2^] / ln(fasting HDL-C [mg/dL]) [20]. In this study, we divided participants into two groups by the optimal cutoff point of METS-IR determined by receiver operating characteristic (ROC) curve analysis: lower METS-IR (≤ 34.58; n = 685) and higher METS-IR (> 34.58; n = 891).

### CAC measurements

All the participants underwent CAC measurements, with coronary CT performed by a 256-slice multidetector CT scanner (Brilliance iCT 256; Philips Medical Systems, Cleveland, Ohio) or a 16-slice scanner (Somatom Sensation 16; Siemens Medical Solutions, Forchheim, Germany), and with calcium scans performed by electrocardiogram-gated dose modulation; more details have been described in detail elsewhere [27, 29]. The CACS was calculated by using a software program (Rapidia 2.8; INFINITT, Seoul, Republic of Korea) and the previously reported Agatston score method [27, 30]. As in previous studies, the presence of CAC was defined as a CACS > 0 [31]. In this study, we divided participants into two groups: non-CAC (CACS = 0; n = 1241) and CAC (CACS > 0; n = 335).

### Statistical analysis

Continuous variables were expressed as the mean ± SD or median (interquartile range), and comparisons between groups were performed using independent-sample T-tests or Mann-Whitney U tests. Categorical variables were presented as frequencies (%), and comparisons between groups were performed using chi-square tests or Fisher’s exact test. Logistic regression models with restricted cubic splines were performed to explore the potential nonlinear relationships between TG/HDL-C, TyG index and METS-IR and CAC. The relationship between the TG/HDL-C, TyG index and METS-IR and CAC was evaluated using logistic regression analysis in different models with adjustments for covariables with clinical importance and statistical significance. Model 1: adjusted for age and sex, Model 2: adjusted for variables included in Model 1 and smoking, diabetes, hypertension, and Model 3: adjusted for variables included in Model 2 and SBP, DBP, TC, LDL-C, creatinine, Hs-CRP, HbA1c. C-statistics derived from ROC curve analysis were used to test the discrimination ability of noninsulin-based markers of IR for CAC. DeLong’s test was performed to compare the area under the curve (AUC) of these markers. All statistical tests were performed with SPSS 19.0 (SPSS Inc., Chicago, Illinois, USA), MedCalc version 19.1 (MedCalc Software, Belgium) and R Programming Language (version 3.6.3). A two-tailed P value < 0.05 was regarded as statistically significant.

## Results

### Baseline characteristics of the study population

The baseline characteristics of the participants were showed in Table 1. The study included 1576 participants (mean age: 52.86 ± 7.05 years; 76.10% men). The participants were divided into two groups based on the optimal cutoff point of METS-IR. Compared with participants in the lower METS-IR group, those with higher METS-IR showed higher SBP, DBP, BMI, TG, LDL-C, Hs-CRP, creatinine, fasting glucose, HbA1c, TG/HDL-C and TyG index, lower HDL‑C, and a higher percentage of male, smoking, diabetes, hypertension and CAC.

**Table 1:** Baseline characteristics of participants stratified by the optimal cutoff point of METS-IR.

	Total population	METS-IR ≤ 34.58	METS-IR > 34.58	P value
N	1576	685	891	
Age (years)	52.86 ± 7.05	53.09 ± 6.98	52.69 ± 7.10	0.256
Male, n (%)	1200 (76.10)	399 (58.20)	801 (89.90)	< 0.001
Smoking, n (%)	231 (14.70)	68 (9.90)	163 (18.30)	< 0.001
Diabetes, n (%)	181 (11.50)	33 (4.80)	148 (16.60)	< 0.001
Hypertension, n (%)	457 (29.00)	143 (20.90)	314 (35.20)	< 0.001
SBP (mmHg)	117.35 ± 13.51	113.91 ± 13.62	120.00 ± 12.83	< 0.001
DBP (mmHg)	77.97 ± 10.30	75.24 ± 10.60	80.06 ± 9.56	< 0.001
Body mass index (kg/m^2^)	24.23 ± 2.70	22.13 ± 1.73	25.85 ± 2.13	< 0.001
Total cholesterol (mg/dL)	195.28 ± 33.85	195.93 ± 32.48	194.78 ± 34.88	0.502
TG (mg/dL)	125.45 ± 71.48	90.21 ± 41.85	152.55 ± 77.49	< 0.001
LDL‑C (mg/dL)	123.35 ± 30.53	120.21 ± 29.82	125.74 ± 30.86	0.001
HDL‑C (mg/dL)	52.05 ± 11.94	59.75 ± 11.83	46.14 ± 8.01	< 0.001
Hs-CRP (mg/dL)	0.05 (0.03, 0.12)	0.04 (0.02, 0.10)	0.07 (0.03, 0.14)	< 0.001
Creatinine ( mg/dL)	0.93 ± 0.18	0.88 ± 0.19	0.97 ± 0.16	< 0.001
Fasting glucose (mg/dL)	101.50 ± 18.97	95.64 ± 13.20	106.00 ± 21.35	< 0.001
Hemoglobin Alc (%)	5.79 ± 0.61	5.66 ± 0.43	5.90 ± 0.70	< 0.001
TG/HDL-C	2.18 (1.40, 3.32)	1.39 (0.97, 1.98)	2.97 (2.08, 4.21)	< 0.001
TyG index	8.61 ± 0.58	8.27 ± 0.48	8.88 ± 0.50	< 0.001
METS-IR	35.85 ± 5.91	30.63 ± 2.88	39.86 ± 4.29	< 0.001
CAC (%)	335 (21.30)	102 (14.90)	233 (26.20)	< 0.001

Data were expressed as mean ± SD, median (interquartile range), or n (%)

*METS-IR* metabolic score for insulin resistance, *SBP* systolic blood pressure, *DBP* diastolic blood pressure, *TG* triglyceride, *LDL-C* low-density lipoprotein cholesterol, *HDL-C* high-density lipoprotein cholesterol, *Hs-CRP* high sensitivity C-reactive protein, *TyG index* triglyceride-glucose index, *CACS* coronary artery calcification score, *CAC* coronary artery calcification

### Associations of the METS-IR, TyG index and TG/HDL-C with CAC

**Table 2:** Univariate logistic regression analysis of the incidence of CAC.

Variable	OR	95% CI	P value
Age	1.056	1.038–1.075	< 0.001
Male	2.344	1.671–3.288	< 0.001
Smoking	1.726	1.343–2.218	< 0.001
Diabetes	3.639	2.634–5.026	< 0.001
Hypertension	2.467	1.919–3.171	< 0.001
Systolic blood pressure	1.019	1.010–1.028	< 0.001
Diastolic blood pressure	1.026	1.014–1.038	< 0.001
Body mass index	1.136	1.086–1.188	< 0.001
Total cholesterol	0.996	0.992–1.000	0.030
Triglyceride	1.003	1.001–1.004	< 0.001
Low-density lipoprotein cholesterol	0.996	0.992–1.001	0.096
High-density lipoprotein cholesterol	0.983	0.972–0.993	0.001
High sensitivity C-reactive protein	1.267	0.855–1.878	0.239
Creatinine	1.915	0.973–3.768	0.060
Fasting glucose	1.023	1.016–1.029	< 0.001
Hemoglobin Alc	1.982	1.635–2.403	< 0.001
Triglyceride/high-density lipoprotein cholesterol	1.116	1.052–1.184	< 0.001
Triglyceride-glucose index	1.899	1.533–2.354	< 0.001
Higher METS-IR	2.024	1.564–2.620	< 0.001

*CAC* coronary artery calcification, *METS-IR* metabolic score for insulin resistance, *OR* odd ratio, *CI* confidence interval

**Fig. 2:**
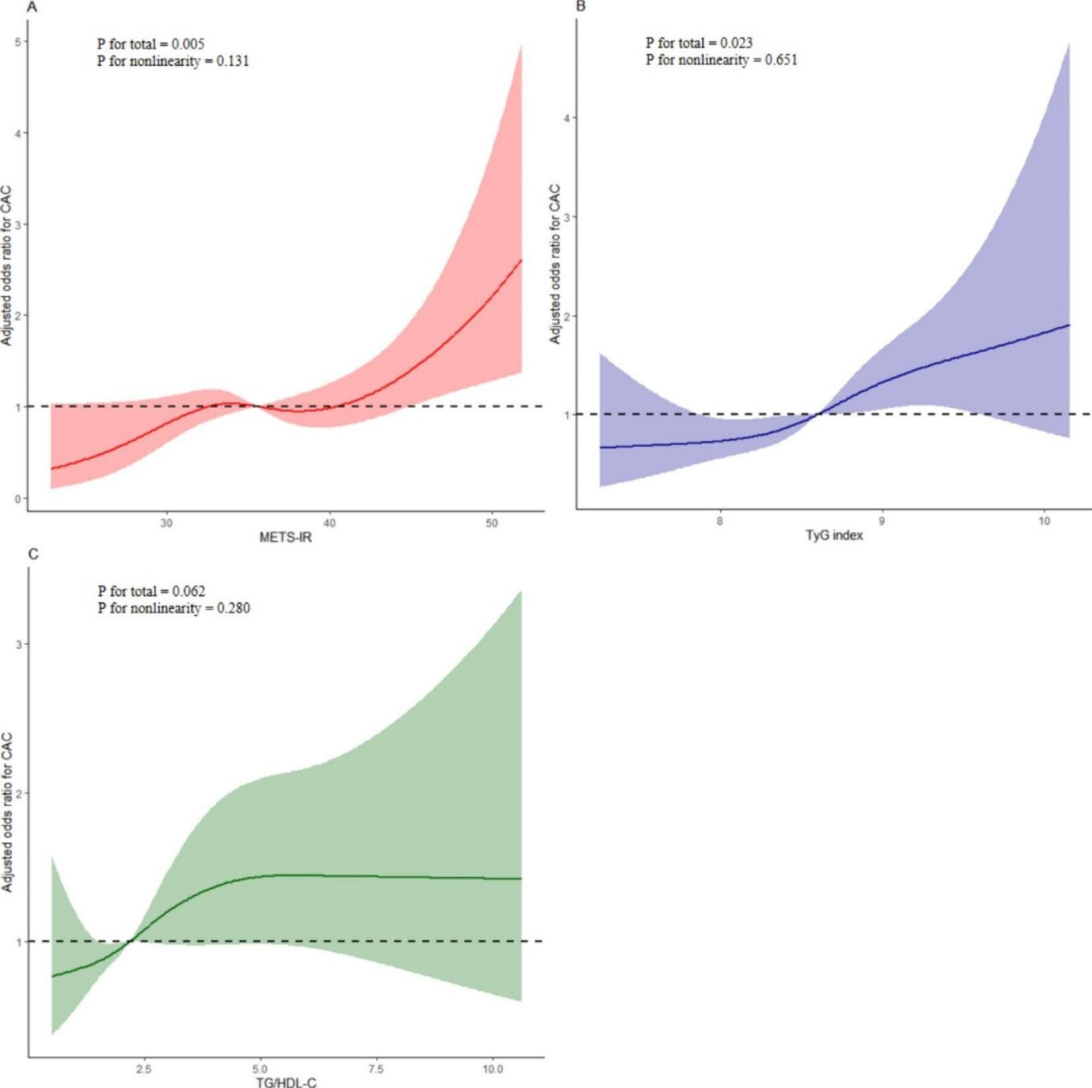
Restricted cubic spline plots of the association between METS-IR (**A**), TyG index (**B**) and TG/HDL-C (**C**) with CAC. The associations were adjusted for age, sex, smoking, diabetes, hypertension, systolic blood pressure, diastolic blood pressure, total cholesterol, low-density lipoprotein cholesterol, creatinine, high sensitivity C-reactive protein, hemoglobin Alc. *CAC* coronary artery calcification, *METS-IR* metabolic score for insulin resistance, *TyG index* triglyceride-glucose index, *TG* triglyceride, *HDL-C* high-density lipoprotein cholesterol

As shown in Table 3, in logistic regression analysis, three models (Models 1–3), including covariables with statistical significance (P < 0.1) and clinical significance in Table 2, were constructed to assess the predictive significance of METS-IR, TyG index and TG/HDL-C for CAC. With the increase in confounding factors, higher METS-IR, TyG index and TG/HDL-C remained independent risk predictors of CAC, despite their being regarded as nominal or continuous variables (all P < 0.05 in Models 1–3). Furthermore, logistic regression models with restricted cubic splines indicated that after full adjustments for the confounders (Model 3), the METS-IR, TyG index and TG/HDL-C were linearly correlated with CAC (all P for nonlinearity > 0.05), and demonstrated that the significance of METS-IR in predicting CAC was higher than that of the TyG index and TG/HDL-C (METS-IR, P for total = 0.005; TyG index, P for total = 0.023; TG/HDL-C, P for total = 0.062; respectively) (Fig. 2).

**Table 3:** Multivariate logistic regression analysis of factors associated with the incidence of CAC

	Model 1		Model 2		Model 3		
	OR (95% CI)	P value	OR (95% CI)	P value	OR (95% CI)	P value	
METS-IR	1.064 (1.040, 1.089)	< 0.001	1.041 (1.017, 1.066)	0.001	1.046 (1.019, 1.073)	0.001	
METS-IR^a^	1.717 (1.304, 2.262)	< 0.001	1.406 (1.057, 1.871)	0.019	1.499 (1.091, 2.060)	0.013	
TyG	1.813 (1.442, 2.278)	< 0.001	1.415 (1.110, 1.804)	0.005	1.439 (1.103, 1.878)	0.007	
TyG^b^	2.079 (1.594, 2.710)	< 0.001	1.591 (1.198, 2.113)	0.001	1.730 (1.264, 2.368)	0.001	
TG/HDL-C	1.105 (1.038, 1.176)	0.002	1.071 (1.004, 1.143)	0.037	1.076 (1.003, 1.155)	0.040	
TG/HDL-C^c^	1.892 (1.443, 2.480)	< 0.001	1.685 (1.275, 2.228)	< 0.001	1.770 (1.301, 2.410)	< 0.001	

^a^ The OR was examined regarding lower METS-IR as reference, ^b^ The OR was examined regarding lower TyG as reference, ^c^ The OR was examined regarding lower TG/HDL-C as reference.

Model 1: adjusted for age, sex

Model 2: adjusted for variables included in Model 1 and smoking, diabetes, hypertension

Model 3: adjusted for variables included in Model 2 and systolic blood pressure, diastolic blood pressure, total cholesterol, low-density lipoprotein cholesterol, creatinine, high sensitivity C-reactive protein, hemoglobin Alc. *CAC* coronary artery calcification, *METS-IR* metabolic score for insulin resistance, *TyG* triglyceride-glucose index, *TG* triglyceride, *HDL-C* high-density lipoprotein cholesterol, *OR* odd ratio, *CI* confidence interval

### Discrimination ability of different models for CAC

**Fig. 3:**
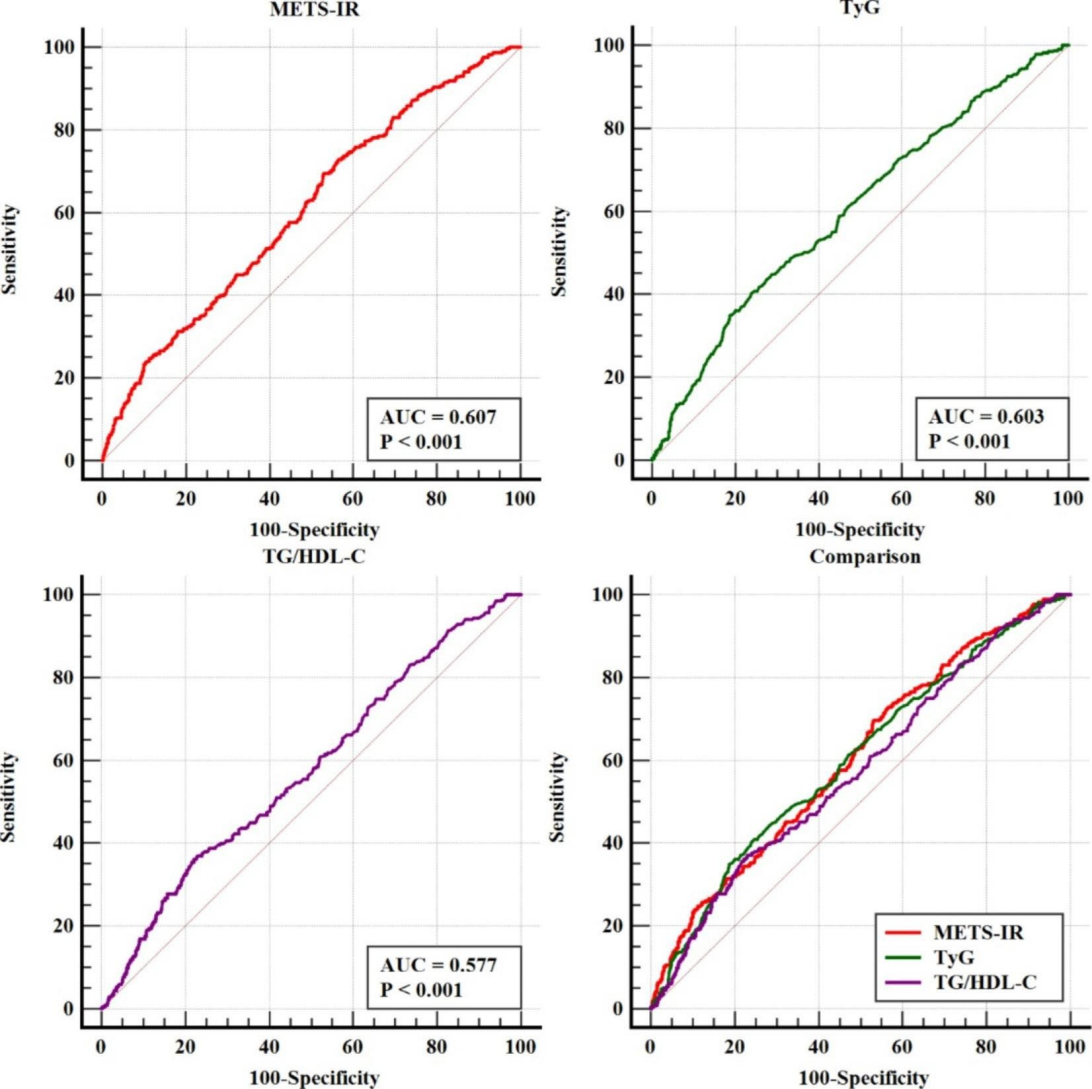
C-statistics evaluating incremental effects of different models. *METS-IR* metabolic score for insulin resistance, *TyG* triglyceride-glucose index, *TG* triglyceride, *HDL-C* high-density lipoprotein cholesterol, *AUC* area under the curve

In ROC curve analysis, the comparative analysis of the AUC of noninsulin-based markers of IR for predicting CAC showed that the discriminant ability of METS-IR and the TyG index were significantly higher than that of TG/HDL-C (all P for comparison < 0.05). In addition, without considering the P value, the AUC of CAC predicted by METS-IR was higher than that of the TyG index, and the addition of METS-IR to a baseline risk model had an incremental effect on the predictive value for CAC (Table 4; Fig. 3).

**Table 4:** C-statistics of discrimination ability of different models for CAC.

variables	AUC	95% CI	P value	Z value	P for comparison
Univariate model					
TG/HDL-C	0.577	0.552–0.601	< 0.001	4.352	Reference
TyG	0.603	0.578–0.627	< 0.001	5.871	0.001
METS-IR	0.607	0.583–0.631	< 0.001	6.243	0.021
Multivariate model					
Baseline risk model^a^	0.692	0.669–0.715	< 0.001	11.531	Reference
Baseline risk model^a^ with TG/HDL-C	0.694	0.671–0.717	< 0.001	11.659	0.486
Baseline risk model^a^ with TyG	0.697	0.674–0.720	< 0.001	11.841	0.257
Baseline risk model^a^ with METS-IR	0.700	0.677–0.723	< 0.001	12.100	0.176

^a^ The baseline risk model included age, sex, smoking, diabetes, hypertension, systolic blood pressure, diastolic blood pressure, total cholesterol, low-density lipoprotein cholesterol, creatinine, high sensitivity C-reactive protein, hemoglobin Alc

*CAC* coronary artery calcification, *TG* triglyceride, *HDL-C* high-density lipoprotein cholesterol, *TyG* triglyceride-glucose index, *METS-IR* metabolic score for insulin resistance, *AUC* area under the curve, *CI* confidence interval

## Discussion

To the best of our knowledge, this cross-sectional study was the first report on the relationship between METS-IR and CAC. In this study, we retrospectively explored the potential association between METS-IR and CAC, and determined that METS-IR had better discrimination ability in predicting the incidence of CAC in asymptomatic adults without CVD than other non–insulin-based IR indexes. The main findings showed a significant positive and linear association between the METS-IR and CAC after adjusting for confounding factors. Moreover, the results also provided evidence that without considering the comparative P value, the predictive significance and discrimination ability of METS-IR for CAC were higher than those of the TyG index and TG/HDL-C, which elucidated that in addition to the TyG index and TG/HDL-C, METS-IR also played an important role in CAC.

IR is defined as the impaired biological response of major tissues such as adipose tissue, muscle and liver to insulin stimulation, which can damage glucose metabolism and lead to compensatory increases in β-cell insulin production and hyperinsulinemia [32]. Although IR has been proven to involve some genetic factors, it is mainly a secondary disease state related to excess body fat, which can lead to many pathological conditions such as dysfunction of blood glucose, blood pressure and blood lipids, visceral obesity, increased inflammatory markers, endothelial dysfunction and prothrombin state [32]. An abundance of studies have shown that IR, which is thought to occur more than 10 years earlier than type 2 diabetes (T2DM), is mainly associated with T2DM [33]; additionally, it is also associated with some metabolism-related diseases, including obesity, metabolic syndrome, arterial stiffness, CAC and CVD [10, 17, 18, 31, 32, 34–36].

Recently, a novel non–insulin-based substitute of IR combining glucose and lipid metabolism and body fat components was developed, called METS‐IR, which has been proven to be superior to the TyG index and TG/HDL-C in the diagnosis of diabetes [20], which was basically consistent with our main findings; that is, METS-IR might be superior to TyG and TG/HDL-C in the diagnosis of CAC. Consistent with a previous study [31], our study also found that a higher TyG index was related to a higher risk of CAC. Intriguingly, our study also obtained an unexpected result, namely, METS-IR may have better diagnostic performance in CAC. In addition, a large-scale epidemiological study involving 142,005 adults showed that in the fully adjusted model, only METS-IR was positively correlated with blood pressure levels (P < 0.001), while the TyG index and TG/HDL-C were not [21]. Similar to this study, Fan J et al. used logistic regression analysis to explore the relationship between TG/HDL-C, TyG index and METS-IR with prehypertension, and found that among the three indexes, after full adjustment, only METS-IR was positively associated with SBP and DBP, and only METS-IR was significantly correlated with prehypertension, regardless of the classification of waist circumference [22]. Moreover, Bello-Chavolla OY et al. also found that compared with TG/HDL-C, TyG index and HOMA-IR, the positive correlation coefficient between METS-IR and pulse wave velocity was higher, and METS‐IR had better discrimination ability for hypertension [23]. In addition, several studies have shown that METS-IR is associated with new-onset diabetes [25], ischemic heart disease [26] and metabolic syndrome [37]. While different from our research, Mirr M et al. have shown that the diagnostic performance of the TyG index and TG/HDL-C was better than METS-IR in metabolic syndrome [37]. The reason for this difference may be due to different research populations and specific diseases. Therefore, the diagnostic performance of these noninsulin-based markers of IR for metabolic related diseases still needs to be further explored in large-scale clinical studies and epidemiological investigations.

In summary, our study found that the METS-IR was better than the TyG index and TG/HDL-C at predicting the incidence of CAC in asymptomatic adults without CAD, which further verified that METS-IR may be used as a simple, low-cost and noninvasive marker to assess the prevalence of CAC. Although the mechanisms of the higher association of the METS-IR than the TyG index and TG/HDL-C with CAC have not been fully clarified, it may be attributed to the fact that METS-IR is superior to the TyG index and TG/HDL-C in evaluating IR. Alternatively, compared to the TG/HDL-C and TyG index, which represented IR in the liver and muscle, the METS-IR, due to the involvement of BMI, might serve as a better indicator of IR in adipose tissue, muscle and liver and a more valuable marker of CAC [38]. In addition, the biological mechanism of the association between METS-IR and CAC is still unclear and may be mediated by the following biological mechanisms. First, IR can promote dyslipidemia, visceral obesity, elevated inflammatory markers, endothelial dysfunction and prothrombin status [39], and then lead to the occurrence of CAC through these pathological mechanisms. Additionally, Ding L et al. also found that METS-IR was positively associated with inflammatory activity and adipokine disorder, all of which may lead to the occurrence and progression of CAC [24]. Second. As mentioned earlier, IR can disrupt glucose metabolism, leading to compensatory increases in β-cell insulin production and hyperinsulinemia, which in turn leads to diabetes and other metabolic diseases closely related to CVD and CAC [40, 41]. For example, Won et al. confirmed that IR, represented by the TyG index, is an independent predictor of CAC progression, especially in adults with less severe baseline CAC [42]. Third, due to the addition of BMI, METS-IR may be a better indicator of IR in muscle, liver and adipose tissue, and may play a more important role in the occurrence of CAC [43, 44].

### Strengths

Overall, this study had several strengths. First, our study described the relationship between METS-IR and CAC for the first time, filling the gap in this research field. Second, our study confirmed the linear correlation between METS-IR and the risk of CAC. Third, we not only found that METS-IR was better than TyG and TG/HDL-C in distinguishing CAC, but also found that it could increase the ability of baseline models including traditional cardiovascular risk factors to predict CAC.

### Limitations

Although this study obtained surprising results, it still had some limitations. First, this study was a cross-sectional study, which could not identify the causal relationship between METS-IR and CAC. Second, this study failed to explore insulin-based IR markers, such as HOMA-IR and QUICKI, and lacked a comprehensive comparison. Third, this study did not include data on anti-dyslipidemic and anti-hypertensive medications, which might miss potential confounding factors. Finally, the data of this study only came from the general population in a single center, so the findings might not be widely applicable. Despite these limitations, this study demonstrated for the first time the robustness of the correlation between METS-IR and CAC in asymptomatic patients without CVD.

## Conclusion

Taken together, the evidence provided by us showed for the first time that the METS-IR was independently related to CAC, and compared with the TyG index and TG/HDL-C, the METS-IR had a stronger ability to distinguish CAC, which elucidated the important role of METS-IR in determining CAC in the general population. The findings supported the importance of controlling METS-IR and its components in reducing CAC risk, and it is of great significance in the actual clinical environment and epidemiological investigation.

## Data Availability

Data can be downloaded from from a public database (https://figshare.com) **Copyright and License Policy** The original data used in this article is authorized by the CC-BY 4.0 license, and the original data has not been changed. After appropriate reference, this article can be exempted from liability. The license link is as follows: https://creativecommons.org/licenses/by/4.0/
